# Standard operating procedures for standardized mass rearing of the dengue and chikungunya vectors *Aedes aegypti* and *Aedes albopictus* (Diptera: Culicidae) - I - egg quantification

**DOI:** 10.1186/s13071-014-0631-2

**Published:** 2015-01-23

**Authors:** Min-Lin Zheng, Dong-Jing Zhang, David D Damiens, Hanano Yamada, Jeremie RL Gilles

**Affiliations:** Insect Pest Control Laboratory, Joint FAO/IAEA Division of Nuclear Techniques in Food and Agriculture, International Atomic Energy Agency, Vienna, Austria; Beneficial Insects Institute, Fujian Agriculture and Forestry University, Fuzhou, Fujian Province China

**Keywords:** *Aedes aegypti*, *Aedes albopictus*, Egg quantification, Mass rearing

## Abstract

**Background:**

Quantification of eggs prior to rearing the immature stages of mosquitoes is an essential step in establishing a standardized mass rearing system. To develop a simple and accurate method of egg quantification for *Aedes aegypti* and *Aedes albopictus*, the relationship between egg number and weight, as well as egg number and volume, were studied.

**Methods:**

Known quantities of eggs (1,000, 3,000, 6,000, 12,000, 15,000, 18,000, 21,000 and 27,000) were counted and subsequently their weight and volume were measured. Best-fit curves and regression equations were used to describe relationships between *Aedes* egg number and both weight and volume.

**Results:**

Eighteen thousand *Ae. aegypti* eggs weighed 159.8 mg and had a volume of 277.4 μl, compared to measurements of 131.5 mg and 230.3 μl for *Ae. albopictus*. The eggs of *Ae. aegypti* were thus larger and heavier than those of *Ae. albopictus*. The use of weight and volume to quantify egg number was validated by counting volumes and weights of eggs expected to correspond to 3,000 and 18,000 eggs of each species; significant correlations were found in all cases except in the case of 3,000 *Ae. albopictus* eggs measured by volume.

**Conclusion:**

Methods for egg quantification were validated and shown to be a consistent and practical means to achieve uniform distribution of *Aedes* larvae between rearing trays, important for optimal mass rearing of the immature stages of *Aedes* mosquitoes.

## Background

The incidence of dengue has grown dramatically around the world in recent decades with over 3 billion people now at risk [1]; the disease is endemic in more than 100 countries in Africa, the Americas, the Eastern Mediterranean, Southeast Asia and the Western Pacific and outbreaks are becoming more frequent. While *Aedes aegypti* remains the primary vector of the dengue virus, *Ae. albopictus* is playing an increasingly important role.

One strategy being pursued to more effectively control these disease vectors Area-Wide Integrated Vector Management (AW-IVM) programmes with a sterile insect technique (SIT) component, which has succeeded in controlling other insect pests of agricultural significance such as fruit flies, tsetse flies, and the New World screwworm [2-6]. Since 2010, the Insect Pest Control Laboratory (IPCL) of the Joint FAO/IAEA Division of Nuclear Techniques in Food and Agriculture, Seibersdorf (Austria) is developing an SIT package for *Aedes* mosquitoes which includes techniques and equipment for mass-rearing, sex separation, and sterilization. A larval rearing unit comprising a rack containing 50 trays [7], high efficiency diet formulations for larval development [8,9] and an adult mass-rearing cage [10] have also been developed for *Ae. albopictus* and are being tested for *Ae. aegypti*.

Development of mass-rearing tools and standard operating procedures (SOPs) are not only useful for the classical SIT using radiation to sterilize the males, it is also a prerequisite for the Incompatible Insect Technique (IIT) using *Wolbachia*-infected males [11,12] and for transgenic population suppression approaches such as RIDL [13,14]. All techniques mentioned above require the production of large numbers of high quality males for sequential releases into the target area to induce sterility into the wild mosquito population. In order to achieve this goal, standardization of all steps of the mass-rearing process is required, starting from egg production. Each tray of the rack/tray system needs to be seeded with the same number of eggs to control and ensure the quality of the final product, avoiding disparities between trays. An accurate quantitative estimation of eggs would allow a consistent distribution of eggs, avoiding underfilling or overcrowding. Underfilling leads to production inefficiency, a waste of rearing diet and potential overfeeding while overcrowding leads to small pupae and adults, prolonged development times and increased mortality [15,16].

In the present paper, we propose two standardized methods for estimation of egg numbers for *Ae. aegypti* and *Ae. albopictus*, based on weight or volume, that could be used for routine rearing or mass-rearing purposes.

## Methods

### Ethics statement

The blood used for routine blood-feeding was collected in Vienna, Austria during routine slaughtering of pigs or bovines in a national authorized abattoir at the highest possible standards strictly following EU laws and regulations.

### Colony maintaining for experiments

*Ae. aegypti* and *Ae. albopictus* laboratory colonies originating from Juazeiro, Brazil and Rimini, Italy, respectively, were used for all experiments. Larvae were reared in plastic trays (30 × 40 × 8 cm) containing 1 liter of deionized water held at a constant air temperature of 27 ± 1°C and a photoperiod of 12:12 (L:D). Larval food comprised 50% tuna meal, 36% bovine liver powder, 14% brewer’s yeast and 0.2 g of Vitamin Mix per 100 ml of diet solution. approximately 4–5,000 adult mosquitoes were reared per 60 × 60 × 60 cm cage (Bioquip, Rancho Dominguez, Ca.) at a constant ambient temperature of 25 ± 1°C, a photoperiod of 12:12(L:D) and relative humidity of ca. 70%. Blood meals were offered to females three times per week and a 10% sugar solution was constantly available.

### Egg collection, drying and storage

Females were allowed to oviposit in a cylindrical container (diameter 11.4 cm, height 9.7 cm, BioQuip, Rancho Dominguez, Ca.) containing deionized water and lined with crêpe paper (Sartorius Stedim Biotech GmbH, Göttingen, Germany). Egg-papers were removed and gently washed with deionized water using a plastic washing bottle to remove dead adults and transferred into a covered larval rearing tray for gradual drying over 24–48 hours at 27 ± 1°C and 70% RH. Egg-papers were transferred into plastic zip lock bags and kept in a sealed black plastic box for maturation in the larval rearing room, at conditions described above. All eggs used in these experiments were collected, stored and treated in the same way, all no older than 15 days post-oviposition.

### Relationship between quantity and weight of eggs

Eggs were brushed from their papers using a small paint brush and collected on a ceramic palette for counting under a stereomicroscope. Batches of 1,000, 3,000, 6,000, 12,000, 15,000, 18,000, 21,000 and 27,000 eggs were transferred into 1.5 ml Eppendorf tubes for weighing using an electronic balance with an accuracy of 0.0001 g. Each size of egg batch was repeated at least 3 times for each species, using different batches of eggs each time.

### Relationship between quantity and volume of eggs

Using the protocol described above, we measured the volume occupied by the different numbers of eggs (1,000, 3,000, 6,000, 12,000, 15,000, 18,000, 21,000 and 27,000 eggs) in Eppendorf tubes. Eggs were removed and the tubes filled with deionized water to the same level and weighed. Volumes were calculated from the density of water: 1 mg of deionized water has a volume of 1 ml, as confirmed in a prior test (data not shown). Measurements of each egg quantity were repeated at least 3 times.

### Validation of the relationship between egg number and weight

To validate the relationship between egg number and weight, 3,000 and 18,000 eggs were weighed out based on the mean weights of eggs estimated in the previous experiment (Table 1), then counted to compare actual with expected numbers. Validation counts were made for 3,000 and 18,00 eggs at least three times each, for each species.

**Table 1:** **Weight (mean ± SE) of** ***Ae. aegypti*** **and** ***Ae. albopictus*** **egg batches**

**Number of eggs**	**Weight of eggs (mg)**		**t**	**P**
	***Ae. aegypti*** **(N)**	***Ae. albopictus*** **(N)**		
1,000	8.9 ± 0.04 (27)	7.7 ± 0.08 (44)	11.37	***
3,000	25.9 ± 0.5 (15)	22.1 ± 0.26 (17)	7.04	***
6,000	51.4 ± 1.04 (7)	44.5 ± 0.39 (7)	6.17	***
12,000	101.8 ± 3.82 (3)	89.3 ± 0.45 (3)	3.25	**
15,000	132.3 ± 2.42 (3)	111.4 ± 0.86 (3)	8.15	**
18,000	159.8 ± 1.93 (3)	131.5 ± 1.13 (3)	12.67	***
21,000	184.6 ± 2.41 (3)	146.3 ± 2.47 (3)	11.1	***
27,000	233.9 ± 2.97 (3)	187.3 ± 2.43 (3)	12.15	***

“N” is the number of replicates; “***” and “**” represent P < 0.001 and 0.001 < P < 0.05, respectively.

### Validation of the relationship between egg number and volume

Deionized water was added to Eppendorf tubes in quantities corresponding to the volumes measured as described above for 3,000 and 18,000 eggs (Table 2), and the volume marked on the tubes. Eggs were added to the Eppendorf tubes up to the mark and were subsequently removed for counting, and the actual numbers of eggs counted were compared to expected numbers.

**Table 2:** **Volume (mean ± SE) of** ***Ae. aegypti*** **and** ***Ae. albopictus*** **eggs measured from different quantities of counted eggs**

**Number of eggs**	**Volume of eggs (μL)**		**t**	**P**
	***Ae. aegypti*** **(N)**	***Ae.albopictus*** **(N)**		
1,000	18.8 ± 0.29 (17)	16.5 ± 0.36 (17)	4.83	***
3,000	50.7 ± 0.63 (15)	45.2 ± 0.72 (12)	5.74	***
6,000	99.4 ± 1.24 (9)	82.5 ± 1.08 (8)	10.15	***
12,000	187.2 ± 2.17 (6)	159.0 ± 1.61 (5)	10.02	***
15,000	231.8 ± 2.53 (5)	196.3 ± 1.97 (5)	11.08	***
18,000	277.4 ± 2.96 (5)	230.3 ± 3.22 (5)	10.75	***
21,000	323.9 ± 3.95 (5)	259.4 ± 6.39 (5)	8.59	***
27,000	422.0 ± 6.34 (5)	336.6 ± 4.96 (5)	10.61	***

“N” is the number of replicates; “***” represents P < 0.001.

To further validate this correlation, volumes of eggs corresponding to those measured above for 3,000 and 18,000 eggs (Table 2) were added to Eppendorf tubes, then counted and actual numbers compared to expected numbers. Validation by each method of 3,000 and 18,000 eggs of each species was repeated at least 3 times.

### Effect of brushing on egg hatch rate

To determine if the brushing protocol affected egg survival, the hatch rate of approximately 500 eggs were placed in hatching solution without being removed from the egg paper (control) and the hatch rate of ~500 eggs removed from egg paper by brushing were compared.

### Statistical analysis

For the relationship between number of eggs, weight and volume, Pearson’s Correlation coefficients were calculated and tested using Minitab 13.32 (Minitab Inc., Pennsylvania). *Ae. aegypti* and *Ae. albopictus* egg batch weight and volume were compared using SPSS 19.0 statistical software (IBM SPSS Statistics, New York) to calculate two sample t tests for independent samples. For validation, a *t* test was conducted to compare the expected mean with the observed number.

Hatch rates were arcsin transformed (√hatch rate) to allow comparison by *t* test and analyzed for an effect of brushing.

## Results

### Relationship between weight and quantity of eggs

**Figure 1:**
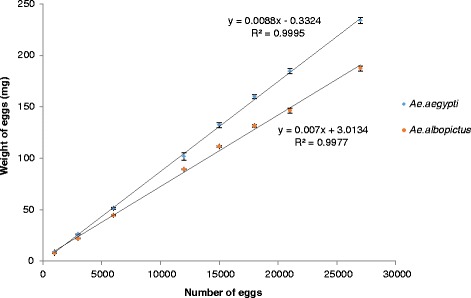
**Relationship between weight and quantity of** ***Aedes aegypti*** **(Blue) and** ***Aedes albopictus*** **(Red) eggs.**

Egg number and weight followed a significant linear relationship in both *Ae. aegypti* and *Ae. albopictus* (y = 0.0087× − 0.0912 with r = 0.99, S = 2.547, P < 0.0001 and y = 0.007× + 1.0317 with r = 0.99, S = 2.090, P < 0.0001, respectively) (Figure 1). The weight of *Ae. aegypti* eggs was shown to be significantly greater than that of *Ae. albopictus* eggs (Table 1).

### Relationship between volume and quantity of eggs

**Figure 2:**
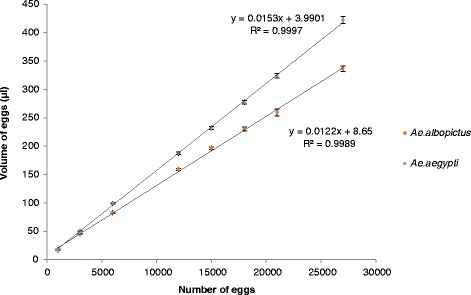
**Relationship between volume and quantity of** ***Aedes aegypti*** **(Blue) and** ***Aedes albopictus*** **(Red) eggs.**

Egg volume and number also followed a significant linear relationship for both *Ae. aegypti* and *Ae. albopictus* (y = 0.0124× + 9.707 with r = 0.99, S = 6.107, P < 0.0001 and y = 0.0155× + 4.59 with r = 0.999, S = 4.393, P < 0.0001, respectively) (Figure 2).

The size of *Aedes aegypti* eggs was shown to be significantly greater than those of *Aedes albopictus* eggs (Table 2).

### Validation of the relationship between egg number and weight

Quantities of *Ae. aegypti* and *Ae. albopictus* eggs expected from the weight experiment above to contain 3,000 (25.9 mg and 22.1 mg, respectively) and 18,000 (159.8 mg and 131.5 mg, respectively) eggs were weighed out and then counted, and the data are presented in Table 3. No significant difference was observed between the expected number of eggs and observed number of eggs, confirming the weighing of dry eggs to be a reliable method for quantifying eggs.

**Table 3:** **The observed number (mean ± SE) of eggs counted from the weight of expected number of eggs for** ***Ae. aegypti*** **and** ***Ae. albopictus***

		***Ae.albopictus***			***Ae.aegypti***		
**Number of eggs expected**		**Number of eggs observed**	**t**	**P**	**Number of eggs observed**	**t**	
3,000	N = 7	3,081 ± 117	1.84	0.12	2,980 ± 134	−0.40	0.71
18,000	N = 3	18,458 ± 623	1.27	0.33	17,780 ± 492	−0.77	0.52

“N” is the number of replicates.

### Validation of the relationship between egg number and volume

Volumes of *Ae. aegypti* and *Ae. albopictus* eggs expected from the volume experiment above to contain 3,000 (50.7 μl and 45.2 μl, respectively) and 18,000 (277.4 μl and 230.3 μ respectively) eggs were measured out and then counted, and the data are presented in Table 4. No significant difference was observed between the expected number of eggs and observed number of eggs except for the volume estimated to be 3,000 eggs for *Ae albopictus*, where significantly more eggs than expected were counted from the measured volume.

**Table 4:** **The observed number (mean ± SE) of eggs counted from the volume of expected numbers of eggs for** ***Ae. aegypti*** **and** ***Ae. albopictus***

		***Ae.albopictus***			***Ae.aegypti***		
**Number of eggs expected**		**Number of eggs observed**	**t**	**P**	**Number of eggs observed**	**t**	
3,000	N = 7	3,172.7 ± 76.9	5.94	<0.005	3,173 ± 233	1.98	0.10
18,000	N = 3	17,452 ± 318	−2.99	0.10	18,164 ± 1180	0.24	0.83

“N” is the number of replicates.

### Effect of brushing on egg hatch rate

For neither species was there an impact of the brushing protocol on hatch rate. Indeed, there was no significant difference between the control (90.8 ± 1.4%) and brushed eggs (89.6 ± 0.7%) (*t* test, t = −1.28, df = 4, P = 0.271) in *Ae. albopictus* or between the control (93.7 ± 1.6%) and brushed eggs (91.9 ± 1.0%) (*t* test, t = 1.65, df = 4, P = 0.175) in *Ae. aegypti*.

## Discussion

A convenient and accurate approach to quantify the dried eggs of *Ae. aegypti* and *Ae. albopictus* has been developed, allowing for standardized larval rearing of *Aedes* mosquitoes. A highly significant correlation between egg number and both weight and volume was observed, and the low variability between replicates indicates a good reproducibility. Considering the management required to produce and coordinate the large quantities of eggs needed in a mass rearing setting, the techniques developed here appear to be more practical on a very large scale than the method previously employed [17] using digital image analysis to estimate *Aedes* egg numbers present on egg papers.

The behavior of *Ae. aegypti* and *Ae. albopictus* reared in the mass rearing cages and developmental trays developed by Balestrino *et al*. [10,18] allow egg production by a colony to be predicted with some accuracy. Indeed the capacity of *Aedes* species to firmly oviposit onto a removable substrate (the oviposition paper) [19,20] and the resistance of eggs to drying [21,22] simplifies the collection and maturation process. For example, a mass rearing cage containing 13,000 adult *Ae. albopictus* allows the harvesting of an egg paper with around 100,000 eggs following one blood meal [10]. Egg papers can then be dried and stored in laboratory conditions. When needed, and because of the robust nature of the eggs, they can be brushed and handled without significant decrease in hatch rate after a storage duration of several weeks (8 to 10 weeks as for *Ae. aegypti* [23,24]) and the quantity estimated.

However, standardized tools need to be developed. In the mass rearing trays (100 × 60 × 3 cm with a capacity of 6 L of water), the optimal quantity of larvae reared in each tray is about 12,000 to 18,000 [7]. Since after drying, storage and brushing, hatching rate of *Ae. aegypti* (Juazeiro strain) and *Ae. albopictus* (Rimini strain) is about 90%, between 13,333 and 20,000 eggs per tray would be needed. Several options could be used to measure and deposit eggs into trays: for example, a measuring spoon sized to contain the desired quantity of eggs could be used to collect and transfer the eggs to trays, or appropriately sized pharmaceutical capsules dissolvable in water could be used to hold and deposit the eggs. Further experiments will deal with the use of hypromellose capsules (Qualicaps® Europe, Spain) of different sizes (No. 4, 5, 6) to obtain the number of eggs suitable for the mass rearing trays.

Although the relationship between quantity and weight or volume of the eggs of two *Aedes* species has been defined here, the application of either of these accurate quantification methods for eggs should be based on a strict, reliable and standardized mass rearing process, since the weight and size of *Aedes* eggs can be affected by several aspects of mass rearing such as egg storage [25,26] as well as the adults’ nutrition or level of health [27].

## Conclusions

Two practical and simple methods of egg quantification were tested and validated. Weight and volume measurements have been shown to be a consistent and reliable means to quantify eggs for transfer to trays to allow uniform rearing of the immature stages of *Aedes* mosquitoes in a mass rearing setting.
